# Development of a supported self-management intervention for adults with type 2 diabetes and a learning disability

**DOI:** 10.1186/s40814-018-0291-7

**Published:** 2018-05-29

**Authors:** Allan House, Gary Latchford, Amy M. Russell, Louise Bryant, Judy Wright, Elizabeth Graham, Alison Stansfield, Ramzi Ajjan, On behalf of the OK Diabetes team, Amanda Farrin, Alexandra Wright-Hughes, Rebecca Walwyn, Claire Hulme, John O’Dwyer

**Affiliations:** 1 Leeds Institute of Health Sciences, University of Leeds, Worsley Building, Leeds, LS2 9NL UK; 2 Leeds Institute of Clinical Trials Research, University of Leeds, Worsley Building, Leeds, LS2 9NL UK; 3 Leeds York Partnership NHS Foundation Trust, Leeds, UK; 4 Division of Cardiovascular and Diabetes Research, University of Leeds, Leeds, UK

**Keywords:** Type 2 diabetes, Supported self-management, Learning disability

## Abstract

**Background:**

Although supported self-management is a well-recognised part of chronic disease management, it has not been routinely used as part of healthcare for adults with a learning disability. We developed an intervention for adults with a mild or moderate learning disability and type 2 diabetes, building on the principles of supported self-management with reasonable adjustments made for the target population.

**Methods:**

In five steps, we:

1. Clarified the principles of supported self-management as reported in the published literature
2. Identified the barriers to effective self-management of type 2 diabetes in adults with a learning disability
3. Reviewed existing materials that aim to support self-management of diabetes for people with a learning disability
4. Synthesised the outputs from the first three phases and identified elements of supported self-management that were (a) most relevant to the needs of our target population and (b) most likely to be acceptable and useful to them
5. Implemented and field tested the intervention

**Results:**

The final intervention had four standardised components: (1) establishing the participant’s daily routines and lifestyle, (2) identifying supporters and their roles, (3) using this information to inform setting realistic goals and providing materials to the patient and supporter to help them be achieved and (4) monitoring progress against goals.

Of 41 people randomised in a feasibility RCT, thirty five (85%) completed the intervention sessions, with over three quarters of all participants (78%) attending at least three sessions.

Twenty-three out of 40 (58%) participants were deemed to be very engaged with the sessions and 12/40 (30%) with the materials; 30 (73%) participants had another person present with them during at least one of their sessions; 15/41 (37%) were reported to have a very engaged main supporter, and 18/41 (44%) had a different person who was not their main supporter but who was engaged in the intervention implementation.

**Conclusions:**

The intervention was feasible to deliver and, as judged by participation and engagement, acceptable to participants and those who supported them.

**Trial registration:**

Current Controlled Trials ISRCTN41897033 (registered 21/01/2013)

**Electronic supplementary material:**

The online version of this article (10.1186/s40814-018-0291-7) contains supplementary material, which is available to authorized users.

## Background

In UK practice, the terms learning disability and intellectually disability are used interchangeably. It is estimated that 2% of the population has a learning disability, with about 1.5% having a mild or moderate disability. Diabetes in people with a learning disability has been estimated to be more common than in the general population [1, 2], with a cited prevalence of 9–11% [3, 4]. People with learning disabilities also have higher rates of hospital admissions from diabetes-related ambulatory care-sensitive conditions [4]. There are a number of possible explanations for high rates of type 2 diabetes mellitus (T2DM) in adults with a learning disability including high prevalence of obesity [5, 6], prescription medications that increase diabetes risk and more limited self-management skills [7].

Supported self-help or self-management with health problems is now well established in that the principles are clear in terms of core elements, although the intensity with which it is delivered and its specific content have varied considerably between studies [8–10]. In relation to intensity, the main variation is in amount of contact with the support/therapist, which ranges from regular face-to-face meetings to limited telephone contact. The usual pattern is that a professional (or trained peer) acts as a ‘therapist’ to help and encourage the nominated patient in using self-management materials.

As regards the content, all programmes contain an educational and an instructional component, the variation residing mainly in the degree of use of formal techniques for supporting change in behaviour and the degree to which they are theory-based. Typical elements [11] include:

- Helping people to understand the short-, medium- and longer term consequences of health-related behaviour
- Helping people to feel positive about the benefits of changing their behaviour
- Building the person’s confidence in their ability to make and sustain changes
- Recognising how social contexts and relationships may affect a person’s behaviour
- Helping plan changes in terms of easy steps over time
- Identifying and planning for situations that might undermine the changes people are trying to make (including planning explicit ‘if–then’ coping strategies to prevent relapse)
- Encouraging people to make a personal commitment to adopt health-enhancing behaviours by setting (and recording) achievable goals in particular contexts, over a specified time
- Helping people to use self-regulation techniques (such as self-monitoring, progress review, relapse management and goal revision) to encourage learning from experience
- Encouraging people to engage the support of others to help them to achieve their behaviour-change goals

Current recommendations for diabetes self-management focus largely on educational and didactic approaches [12]. Development of self-management material for adults with a learning disability has tended to take the same approach [13]. In these programmes, there is typically less emphasis on more autonomous aspects of self-management such as advice about self-monitoring. There is also little on the interaction between the person with diabetes and others supporting their care. Many adults with a learning disability do not live entirely independently even when living in the community—that is, not living in a hospital setting. Family members and other informal or formal carers often provide support in the form of help with shopping, cooking, monitoring and prompting about medication and so on. Living and support arrangements are diverse [14, 15]; some adults with a learning disability remain in the parental home; some live with a sibling or other relative; some live alone or in shared accommodation with non-resident support or peer support from those with whom they share, and some are married or cohabiting with somebody who may or may not themselves have a learning disability. Since many of the positive and negative influences on good diabetes management reside in the immediate social network [16–20], self-management needs to involve not just the person with diabetes but their supporter, and flexibility is needed in negotiating and implementing an intervention.

There is a clear need to improve on this state of affairs. The Equality Act 2010 sets out the legal requirement for public services to provide reasonable adjustments at both service level and individual level for people with a disability, and that should include provision of accessible therapeutic support. This will require modification of even well-established interventions since people with a learning disability have by definition a significantly reduced ability to understand new or complex information and manage independently [21]; many also have communication difficulties. It will involve finding a form of communication that matches the person’s needs and may well involve a supporter who is familiar with helping that person make decisions [22, 23].

We have recently completed a feasibility RCT of a supported self-management intervention for evaluation in a feasibility RCT, the OK Diabetes trial. The protocol for the OK Diabetes RCT [24] and the results for the trial [25, 26] have been reported elsewhere. The present paper reports on our work in developing and field testing the intervention for use in the trial.

The approach we adopted in developing the intervention lies somewhere between the creation of a new therapy on the one hand and standardising (for example in a manual) an existing one. It involved using the principles of an existing therapy in developing the form and content of the intervention, with reasonable adjustments made so that it was suitable for use with our target population. This approach responds to the need to make reasonable adjustments to healthcare interventions for those with a learning disability [27] without sacrificing the effective components of a complex intervention that has been developed and evaluated in the general population.

## Methods

**Fig. 1:**
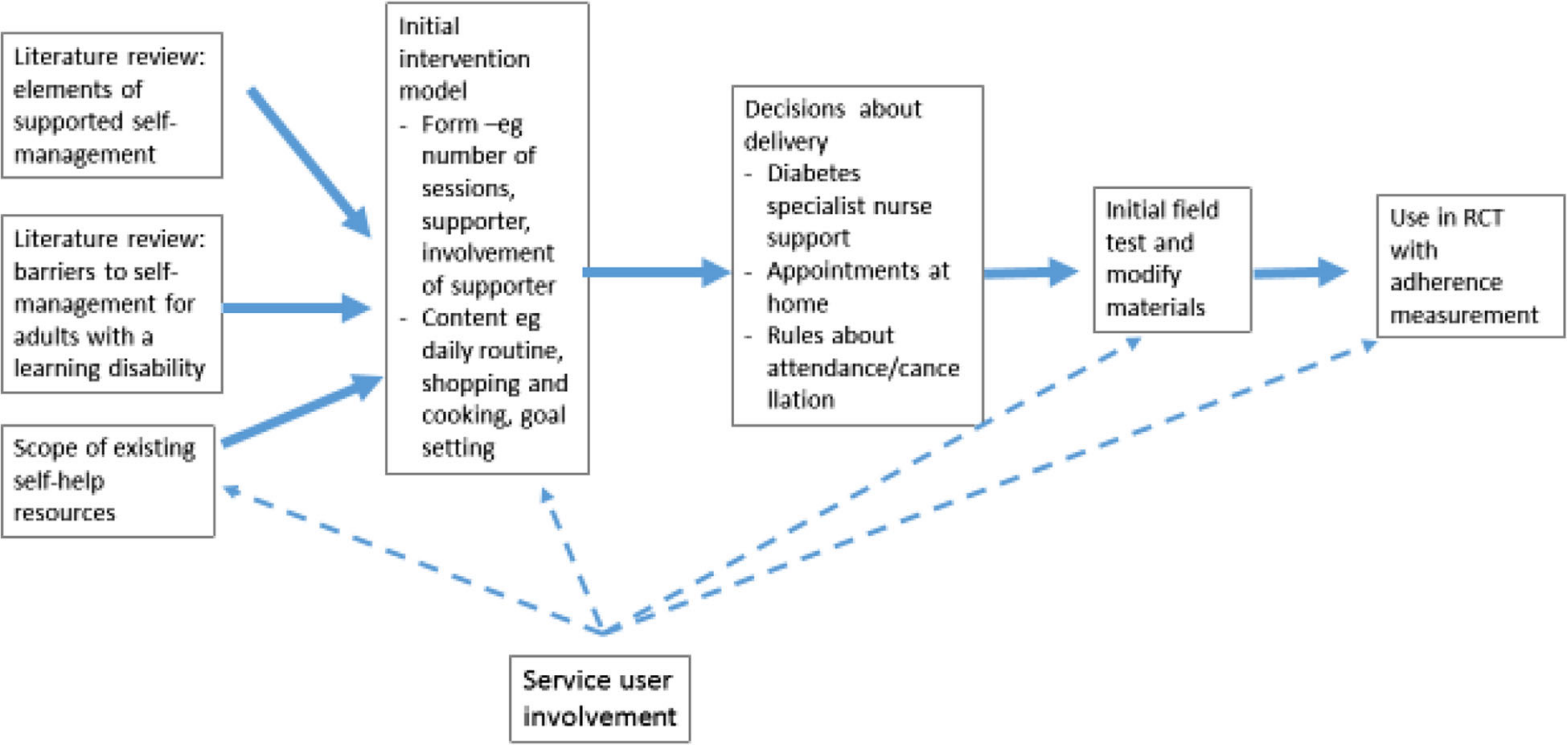
The intervention development process

Supported self-management is an approach to chronic disease management in which the underlying theory and the basic principles of implementation are already established. Development work in the current project did not therefore need to start from the early phases of intervention development (Fig. 1) as recommended by, for example, the UK’s Medical Research Council [28]—elaborating theory and undertaking early phase proof of concept and efficacy studies. Instead we planned our development work in five phases:

1. Clarifying the principles of supported self-management—as summarised in review articles and educational pieces and as identifiable from the protocols and final reports of individual studies of self-management in diabetes
2. Identifying from published literature the reported barriers to effective self-management of type 2 diabetes in adults with a learning disability
3. Collaborating with services that provide health support to adults with a learning disability to review existing materials that aim to support self-management of diabetes for people with a learning disability for examples of good practice
4. Synthesising the outputs from the first three phases to decide on those elements of supported self-management that are most relevant to the needs of our target population (that is, will overcome likely barriers) and most likely to be acceptable and useful (that is, match identified good practices). This phase involved a series of problem structuring and consensus meetings in the research team, at each step checking interim outputs against guidelines on reasonable adjustments and consulting with experts and service users
5. Implementation and field testing of early versions—modifying the intervention materials in the light of feedback from research diabetes nurses supporting the intervention (see later)

At each stage, there were regular consultation meetings involving members of the research team, people with a learning disability and their representatives in local third sector organisations and expert clinicians experience working in learning disability.

We worked with two local third sector organisations with a special interest in supporting people with a learning disability: Tenfold (http://www.tenfold.org.uk/) and People in Action (http://peopleinaction.org.uk/) and with *easy on the i,* (www.easyonthei.nhs.uk) an organisation with special expertise in preparing accessible materials for people with a learning disability. Each group had established relationships with adults with a mild or moderate learning disability, with whom they worked to review materials and policies. Over 12 months, we were introduced to service users who could advise us, by the organisations for which they worked; we met two to three times for each part of the project. Exchanges were not formalised, and we did not record the meetings for research purposes, except to record the advice received.

### Phases 1 + 2: literature reviews

At the time of our study, there were no relevant RCTs in the area of diabetes self-management in learning disability and therefore we did not undertake a formal systematic review of effectiveness studies.

We aimed to use the published literature to identify (a) the principles of self-management that we would hope to embody in our own intervention and (b) those influences on self-management potential in our population that should inform the form or content of a supported self-management programme.

To help identify published information relevant to our intervention, an information scientist (JW) undertook initial searches in four main areas:

- Supported self-management in chronic disease;
- Self-care in diabetes, including barriers to effective self-care;
- Diabetes and a learning disability—a broad search to identify factors that might be specific to the target population;
- Descriptions of specific interventions aimed at improving diabetes control in adults with a learning disability.

Searches were refined in a series of iterative discussions based upon outputs from preliminary searches. We also checked the reference lists of two relevant NICE Guidelines, [11, 29], two Cochrane Reviews, [30, 31], a guideline on supported self-management published by Diabetes UK, [32], and outlines of national standards in diabetes management from the US [33] and the UK [34].

On the basis of these searches, run in MEDLINE, EMBASE, CINAHL and PsychINFO, we identified and reviewed 707 titles and abstracts on the topics of self-management of chronic disease including diabetes, and 350 titles and abstracts on the topic of diabetes in adults with a learning disability.

The searches are available in the full report of the study [26].

Titles and abstracts were reviewed by two of the applicants (AH and GL), and full versions of relevant papers were obtained. We categorised retrieved papers as follows:

- Reviews of self-management and diabetes (*N* = 10) [9, 10, 35–42]
- Individual self-management programmes or interventions (*N* = 22) [43–64]
- Research protocols describing individual self-management interventions (*N* = 8) [65–72]
- Observational studies reporting influences on self-management—barriers and enablers (*N* = 31) [16–20, 73–99]

We extracted data that described the form or content of self-management using an initial framework derived from reviewing the two NICE Guidelines, two Cochrane Reviews and the guideline on supported self-management published by Diabetes UK. We then refined this framework by reference to six individual studies for which reasonably comprehensive descriptions of the intervention were contained in the reports [51, 58, 100–103]. The remaining studies were reviewed against this framework to identify any missing themes, using a modified best-fit framework analysis [104].

Next, we reviewed papers describing barriers to effective self-management, influences on interventions or outcomes that were specific to adults with a learning disability. In a series of review meetings, we organised the identified influences into a descriptive framework. We did not apply a quality assessment to papers, as long as they contained a usable account of intervention components.

Finally, we combined the two frameworks in a synthesis that allowed us to identify the general principles to be adhered to in implementing a pragmatic and sustainable programme of supported self-management, and the form and content of the specific intervention for this project.

### Phase 3: scoping existing resources

For existing self-care resources, a scoping exercise was conducted in which examples of good practice in interventions around health in people with a learning disability were sought from services in the UK. Sources included charities including Diabetes UK, local NHS Trusts and patient groups, NHS Choices and Easyhealth.

We identified 18 examples of resources developed in the UK to support self-management of diabetes for people with a learning disability. The resources were all in leaflet or booklet form, using more-or-less easy-read language, illustrated with photographs or cartoons and covering topics like foods to eat and avoid, exercise, foot care and what to do if ill.

Resources were reviewed by a panel consisting of members of the research team, a dietician in learning disability services and service user representatives. Panel members rated each resource from 1 to 10 on a Likert scale. In informal group discussions that reviewed each resource in turn, members identified which aspects they liked and disliked about each resource (for example, information complexity, font size, use of images) and noted any implications for the new intervention. Results were recorded on a structured proforma.

### Phase 4: problem structuring and priority setting

Using the general approach of problem structuring and priority setting [105], preliminary versions of the supported self-management package—including not just format and content but tailoring (for easy reading, visibility for those with poor acuity and so on)—were discussed in the research team. Finally, we considered guidance on reasonable adjustments to healthcare designed to ensure access for people with a disability, to check that we were meeting these obligations.

A further round of more focused (purposive) reviewing of literature was used to clarify which were the key principles for this population. We generated two checklists derived from the literature and our discussions (see Tables 1 and 2 below) to ensure that all relevant topics were covered, that all components of the intervention could be linked to principles of self-management, and to help frame final decisions (Table 3).

**Table 1:** Elements of supported self-management for type 2 diabetes

What self-management of diabetes involves	
○ Food—buying, preparing, eating	
○ Weight control or weight loss	
○ Physical activity or exercise	
○ Looking after your body—foot care, dental care	
○ Healthy living—alcohol, smoking	
○ Taking tablets	
○ Visiting professionals—dental care, medical care, eye checks	
○ Maintaining emotional wellbeing	
Components of self-management programmes	
○ Education—about diabetes and what it is; what self-management involves	
○ Problem solving	
○ Goal setting, planning	
○ Monitoring and feedback, e.g. blood glucose, weight, dietary intake, tablets take	
○ Skills development—foot care, self-monitoring of blood glucose, preparing food, use of IT	
○ Effective use of other people and resources, e.g. company when going swimming/walking	
○ Managing emotions and building confidence	
Format: what does ‘supported’ mean?	
○ Written materials	
○ Charts—fridge door charts, ‘plan your plate’, diaries	
○ DVD	
○ Web-based programmes—static or interactive/moderated	
○ Telephone or SMS contact—prompts or interactive	
○ IT—beeping fridges, watches, tablet boxes, smart phones, etc.	
○ Groups, e.g. nurse-led, third sector, exercise group, group education	
○ Professional contact—nurse, diabetes educator, GP,	
○ Peer support—informal, trained peer support, family, couples work	
Tailoring of content and format	
○ Literacy and other intellectual attainment	
○ Sensory impairments	
○ Language difficulties—non English, comprehension or speech problems	
○ Self-nominated goals or problems	
○ Professionally identified priorities	
○ Living arrangements	
○ Supporter’s priorities

**Table 2:** Checklist of possible needs and barriers to good healthcare requiring adjustment

Example of impairment or deficit	Example of adjustment (enabler of good healthcare)
Intellectual disability/reduced mental capacity	Staff training in capacity assessment and inclusive practice
Memory problems	Prompts, support for appointments
Literacy/reading skills deficit	Accessible materials, communication skills
Vision/hearing impairment	Visual aids
Speech problems	Time, trained staff
Mobility difficulties, physical symptoms or restrictions	May need OT/physio assessment/mobility aids
Attitudinal barriers	
History of lack of dignity/respect in services	Staff training
Threat to safety including bullying	Safeguarding protocols
Overcoming stigma	Advocacy
Instrumental barriers	
Transport to services	Funding, safe provision
Finance	Personal budget
Lack of access to personal pleasure/R+R activities	Planning meeting with supporter
Treatment burden—timing, side-effects	Support with adherence, modified regime
Social barriers	
Lack of social support/networks	Identify, train and support carers; advocacy; third sector
Talking with professionals	Staff training + supervision
Communicating needs	LD register; Health Action Plan
Understanding—health risks, necessary actions	Accessible information
Low self confidence	Social engagement activities
Mental Health	
Challenging behaviour	Pacing of change; staff training
Distress + mental disorder	Mental health review with learning disability team

**Table 3:** Links between components of OK Diabetes intervention and principles of self-management

Behaviour change: the principles of effective interventions [29]	
Principle	Place in intervention programme (see Additional files 1, 2 and 3)
Helping people to understand the short-, medium- and longer term consequences of health-related behaviour	Review with participants—‘Looking after my diabetes’
Helping people to feel positive about the benefits of changing their behaviour	Discuss plan for change in general terms—‘I am going to…’
Building the person’s confidence in their ability to make and sustain changes	Encourage positive action planning
Recognising how social contexts and relationships may affect a person’s behaviour	Review participant’s life—social network, named supporter and helpers
Helping plan changes in terms of easy steps over time	Make a weekly plan
Identifying and planning for situations that might undermine the changes people are trying to make (including planning explicit ‘if–then’ coping strategies to prevent relapse)	Build ‘if–then’ thinking into action plan
Encouraging people to make a personal commitment to adopt health-enhancing behaviours by setting (and recording) achievable goals in particular contexts, over a specified time	Write goals on visible board
Helping people to use self-regulation techniques (such as self-monitoring, progress review, relapse management and goal revision) to encourage learning from experience	Identify personal rewards for success
Encouraging people to engage the support of others to help them to maintain their behaviour-change goals	Supporter pack and flash cards

Principles for priority setting were:

- The intervention should respond to known barriers to self-management reported by people with a disability—including practical problems such as transport, likely attrition from drop out when multiple attendances are expected, inability to accommodate the presence of a supporter;
- The format of the intervention should be likely to encourage self-maintained change beyond an early supported element; in our target population, this meant especially that the intervention should involve supporters involved with any aspect of lifestyle (shopping, food choice, physical activity, medication monitoring and so on) relevant to diabetes;
- The intervention should be designed to be readily integrated into usual healthcare provision in the NHS, to ensure sustainability.

Based upon all the advice we received from those working with our target group, we wanted to give particular salience to:

- Practical aspects of self-care—buying and preparing food. changes aiming for a healthier balanced diet, increasing physical activity
- Use of simple (accessible) written materials and charts
- Supportive contact both with a professional and with a supporter if one could be identified
- Use of practical goal setting, planning to meet goals and self-monitoring

By contrast, we decided that less helpful aspects would be:

- Education of a more theoretical sort about the nature of diabetes, food values and so on. All participants received factual information about managing their diabetes in a booklet as part of the Treatment As Usual arm of the RCT (https://www.diabetes.org.uk/about_us/news/learning-disabilities-leaflet)
- IT-based interventions, web, DVD, mobile phone etc., because this is usually not readily accessible by our participants
- Group-based interventions—attendance is typically poor and meeting the specific individual needs arising in a heterogeneous population is harder

### Phase 5: implementation and field testing an early version

We decided that professional support would be provided by diabetes nurses with experience in primary care rather than learning disability nurses, the rationale being that (i) very few of the target population would be in contact with (or taken on by) specialist learning disability services; (ii) in routine NHS practice where 20–25% of diabetes patients would be using insulin, diabetes nurses would have more relevant experience; (iii) for our population (not all of whom would have been told they had, or would self-describe as having, a learning disability), the diabetes background was considered to be more acceptable; and (iv) the intervention would be more readily easily used by staff in mainstream services. We planned for the sessions to be delivered in people’s homes to ensure adherence and to allow assessment of the person’s everyday environment, since we did not think the participants would be able to give a good account in a clinic of the influences on their diet, physical activity and self-care.

We developed a training plan for the nurses delivering the intervention covering the underlying principles of mental capacity and of self-management, the individualised elements specific to learning disability, trouble-shooting and dealing with potential problems and the details of the programme and the materials provided with it.

Explicit links were made between the practicalities of the intervention and its rationale in self-management principles (Table 3). We interviewed two general practitioners, a consultant physician in diabetes and a diabetes nurse manager, about any challenges in fitting the intervention into routine care.

The training programme was delivered by two of the researchers (AH, GL) over three sessions of face-to-face contact with the nurses. Both trainers were clinicians (liaison psychiatrist, AH, clinical psychologist GL) with experience of NHS practice in the management of long-term physical illness and previous experience in applied health research and supervision of therapies. We did not involve service users in the training. An additional session on mental capacity assessment was delivered by AS to the nurses and all research interviewers.

Supervised use of the intervention with three initial cases was also arranged. In each case, the whole intervention was delivered over a maximum of four visits and the nurses met together with AH and GL after each visit, to discuss any challenges with implementation. On the basis of this experience, early versions of the intervention were modified in format to make them easier to use. In particular, the forms for keeping notes on each contact were found to be over-structured by the nurses and intrusive for use in the field. Once they had familiarised themselves with the principles of self-management and the nature of each contact, the nurses preferred to make free-form notes during contact and then check afterwards that they had recorded all the necessary information and completed the essential standardised forms (CRF) for the trial.

Both nurses involved in the field-testing reported during case supervision finding the materials easy to use and the nature of the intervention easy to understand.

### Results (see Additional files 1, 2 and 3)

The final intervention had four standardised components with associated materials. How they were delivered depended on participant and supporter characteristics and preferences:

1. Establishing the participant’s daily routines and lifestyle: This included current diet and activity routines, participation in daytime social activities or work, shopping and food preparation, current self-reported health and self-management. The main aim of this component was to identify the social and personal influences in the life of the person with diabetes that would limit their ability to self-manage or that might be mobilised as a resource in supporting self-management.
2. Identifying all supporters and helpers and their roles: A key supporter and other helpers were identified where possible. Key supporters and other helpers were given written information about the project, and if they agreed to support a goal set by the participant, they were given a written reminder of their role. The main aim was to identify people who might be a useful resource in supporting self-management and to ensure any changes were embedded in the social network for longer term maintenance of change.
3. Setting realistic goals for change: The main aim was to avoid prescribing change in the way of good dietary practice or other lifestyle change, but to support goals suggested by the person with diabetes that were specific, simple and achievable given the person’s current routines and social support, and consonant with their willingness to make change. The intention was to encourage engagement in a population usually thought of as having little agency and to introduce the idea of selectable elements in a repertoire of self-management options.
4. Monitoring progress against agreed upon goals: We devised a simple system that did not depend on high levels of functional literacy, using tear-off calendar sheets on which participants noted goal attainment in a Yes/No format. The main aim was to encourage active participation in an activity that is a core feature of self-management.

We prepared materials to accompany these activities:

- For the nurses—templates for a weekly timetable, a chart to record friends and family and other helpers, charts to be completed in collaboration with the person with diabetes (‘my life’, ‘my likes and don’t likes’, ‘looking after my diabetes’)
- For the person with diabetes—an OK Diabetes board to place in a prominent position at home with visible record of goals including pictorial prompts, e.g. ‘snack swaps’, a written action plan in multiple formats and tear-off slips to record daily actions
- For supporters and helpers—an information sheet explaining the study and a card summarising what their role was in helping to support the person with diabetes in meeting their chosen goals

The research nurse worked through the elements of supported self-management with the participant, explaining how to use materials and suggesting initial actions and activities. Further contact was negotiated with the person with diabetes. We anticipated that a total of three to four meetings of 30 to 60 min over 6 to 8 weeks would be provided, followed by telephone support and advice.

We took steps to ensure consistency in the use of the supported self-management: (i) training and supervision sessions with research nurses, (ii) annotation of the intervention materials by research nurses and (iii) ensuring nurses had other experience and training in diabetes or learning disability care prior to the RCT.

## Results

In developing the intervention, we were mindful of the need to assess fidelity (how much the intervention was delivered as planned) and adherence (how successfully the intervention was taken up by participants). We developed a simple approach to collecting intervention materials that could then be used to assess these aspects of intervention performance, the details of which have been reported elsewhere [25, 106].

## Discussion

Although not described in the same detail, similar approaches have been used previously in adapting established and effective therapies to develop brief psychological interventions for depression in people with a learning disability [107, 108].

Our approach to intervention development was based upon the principle of making reasonable adjustments to an existing approach and has a number of advantages. Firstly, because it involved considerable service user and expert input, it proved acceptable to participants. Secondly, its flexibility, which includes when, where and by whom is it delivered, makes our findings transferable to other settings.

As others have noted however [109], even extensively documented and theory-driven approaches to intervention development will entail a degree of personal judgement and will depend upon the particular perspective of the service users and third sector organisations consulted. We are aware for example, that while we privileged an understanding of the individual’s lifestyle and relationship with supporters over psychological characteristics like motivation or knowledge, others have chosen to adapt existing educational programmes for use with this population [110].

## Conclusions

Existing evidence-based interventions can be successfully modified for use with adults who have a learning disability, using literature reviews, service user and expert input to decide upon principle focus and upon desirable form and content of the eventual package. Services can be delivered by healthcare staff with limited experience of LD with relatively little training. Flexibility will always be needed to respond to the differing living and personal arrangements of participants (for example the nature and involvement of a supporter) and variable cognitive abilities.

## Additional files

Additional file 1: Adherence to intervention checklist. (DOCX 178 kb)

Additional file 2: How to sheet: Snack swaps. How to: eat more fruit. How to: be more active. How to: eat more vegetables. (DOCX 7684 kb)

Additional file 3: Intervention materials. (DOCX 209 kb)
