# Supplementary figures and images for: Development of a supported self-management intervention for adults with type 2 diabetes and a learning disability
Source: Pilot Feasibility Stud. 2018 May 29;4:106. doi: 10.1186/s40814-018-0291-7 (PMC5975532; doi:10.1186/s40814-018-0291-7)

# Appendix: Adherence to intervention checklist


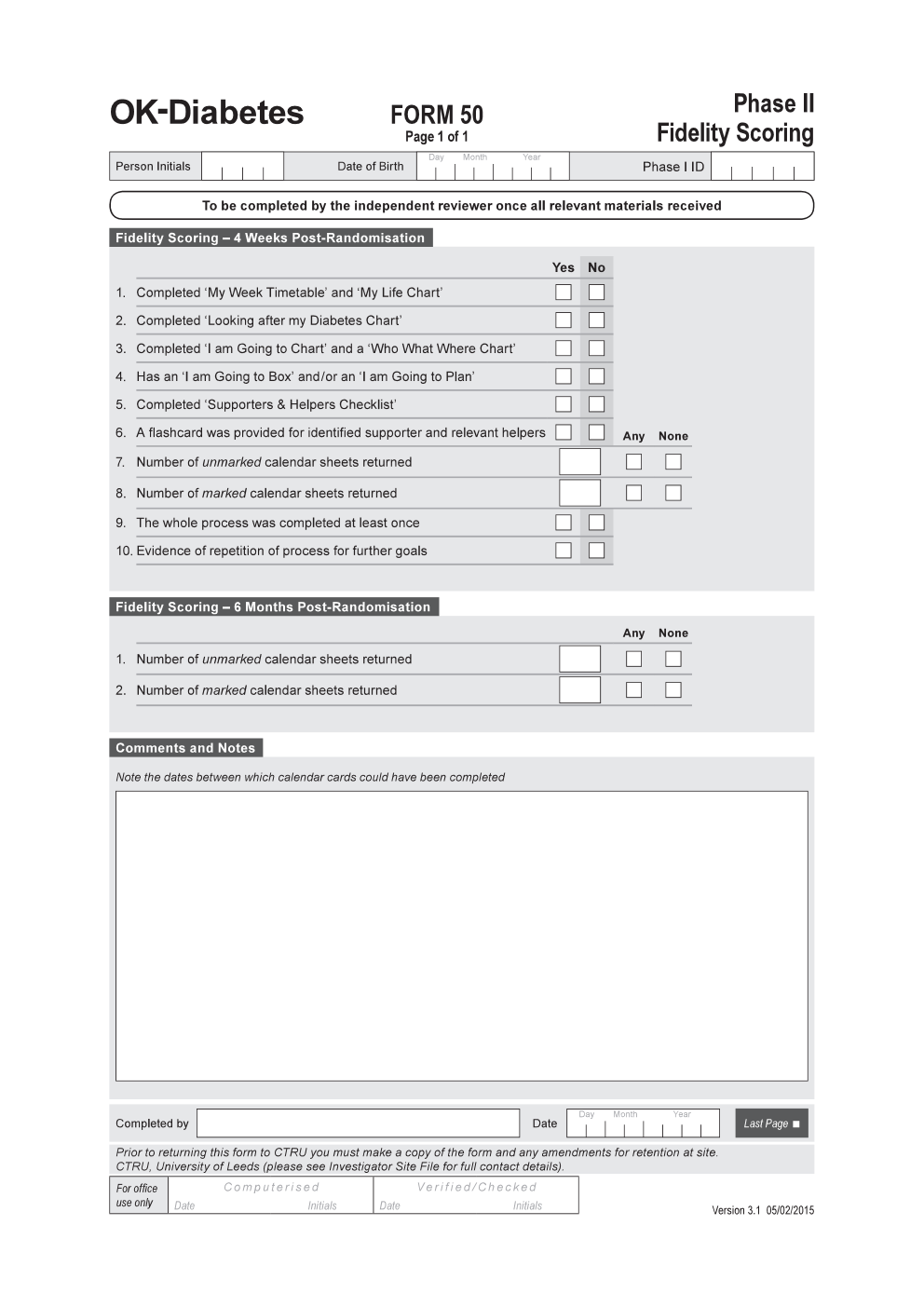

Supplement: Supplementary file 1 — Adherence to intervention checklist. (DOCX 178 kb) [file 40814_2018_291_MOESM1_ESM.docx]
