# Supplementary material for: Development of a supported self-management intervention for adults with type 2 diabetes and a learning disability
Source: Pilot Feasibility Stud. 2018 May 29;4:106. doi: 10.1186/s40814-018-0291-7 (PMC5975532; doi:10.1186/s40814-018-0291-7)

# Appendix: How to sheet: Snack swaps


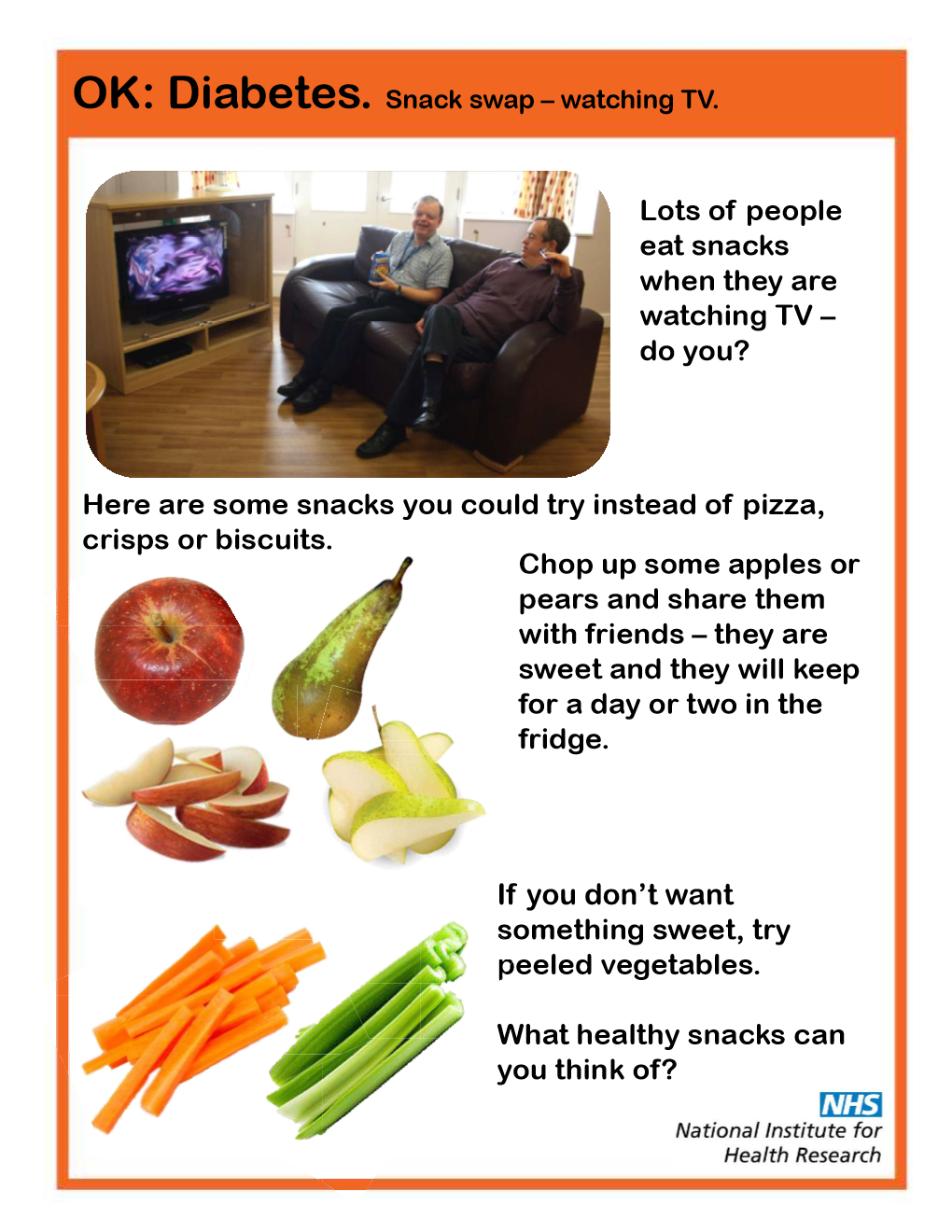


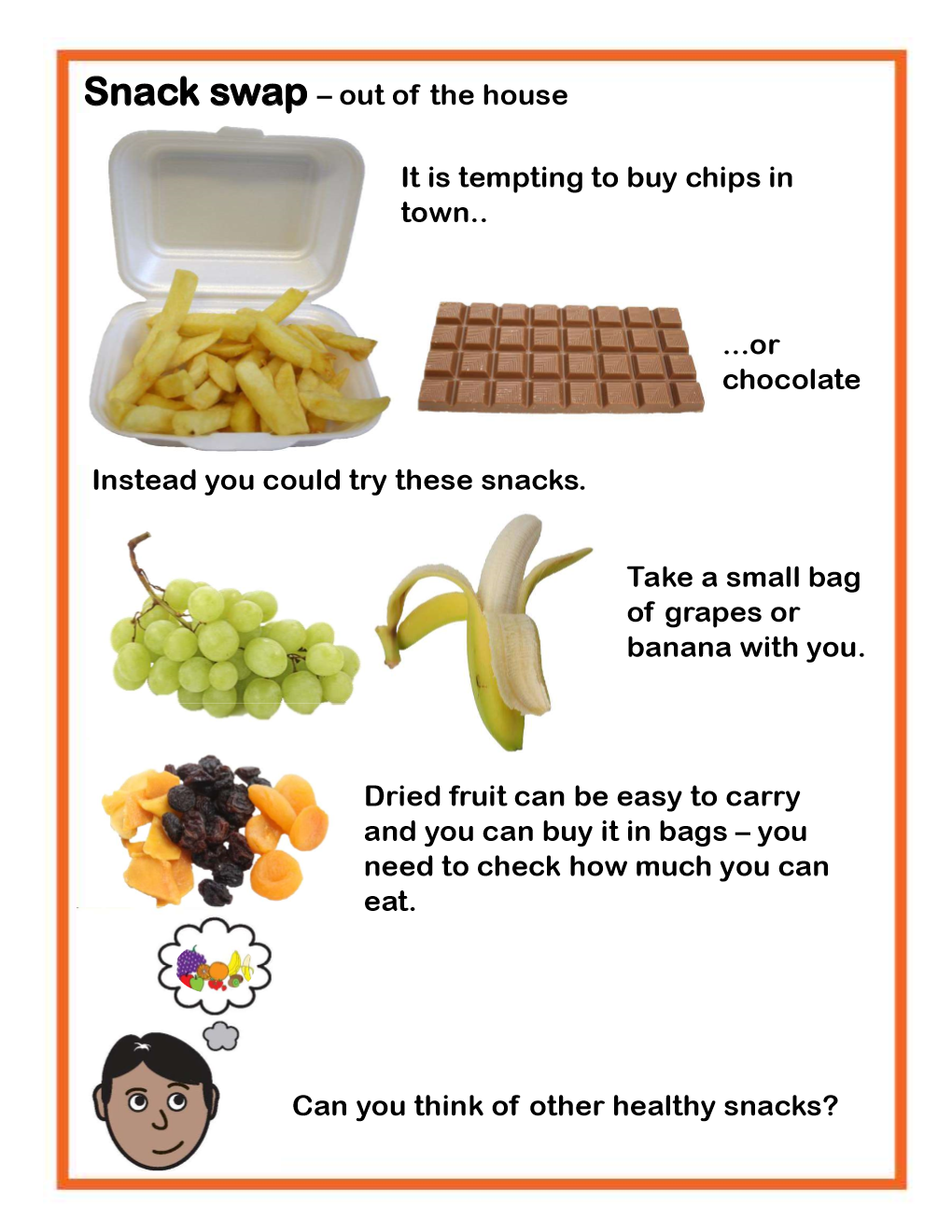


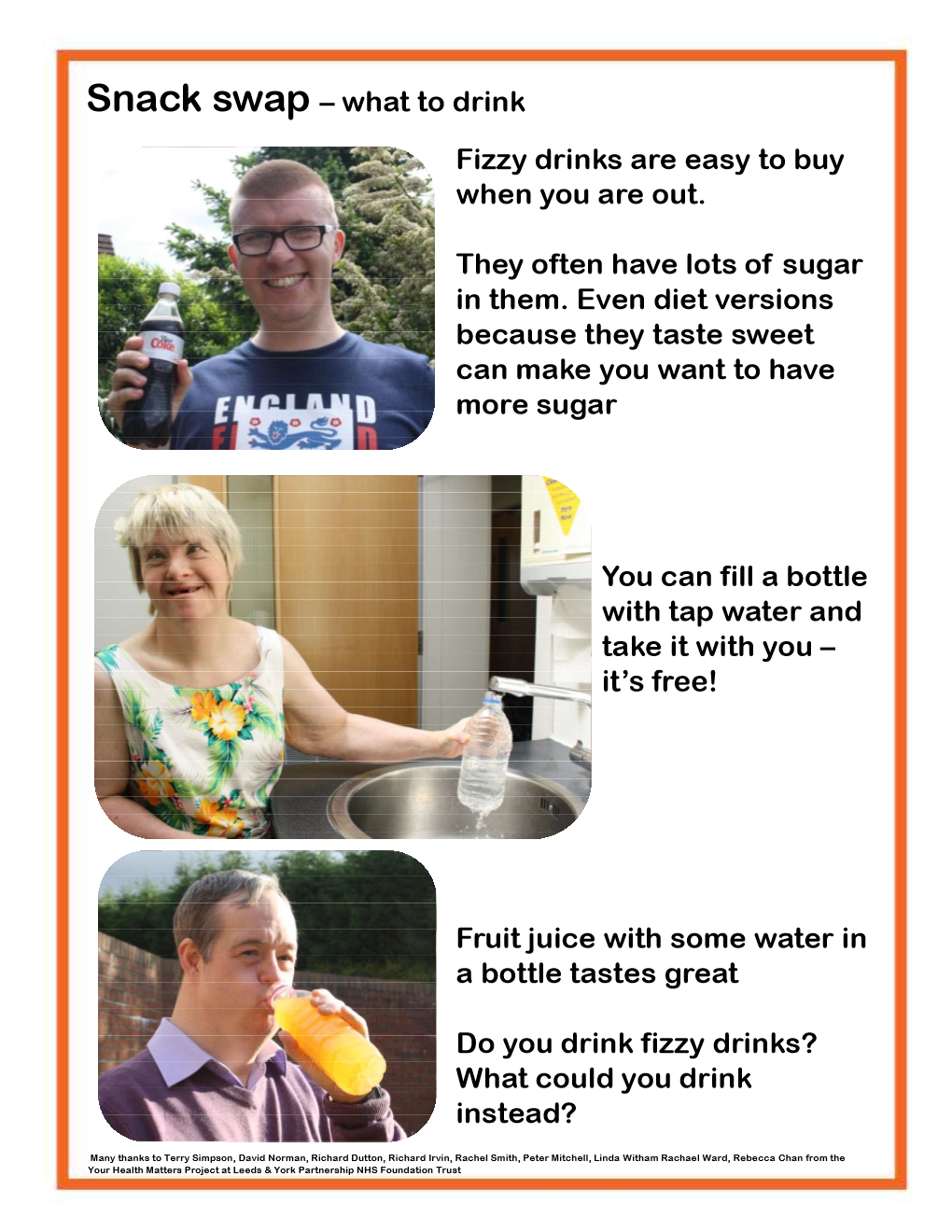


# Appendix: How to: eat more fruit


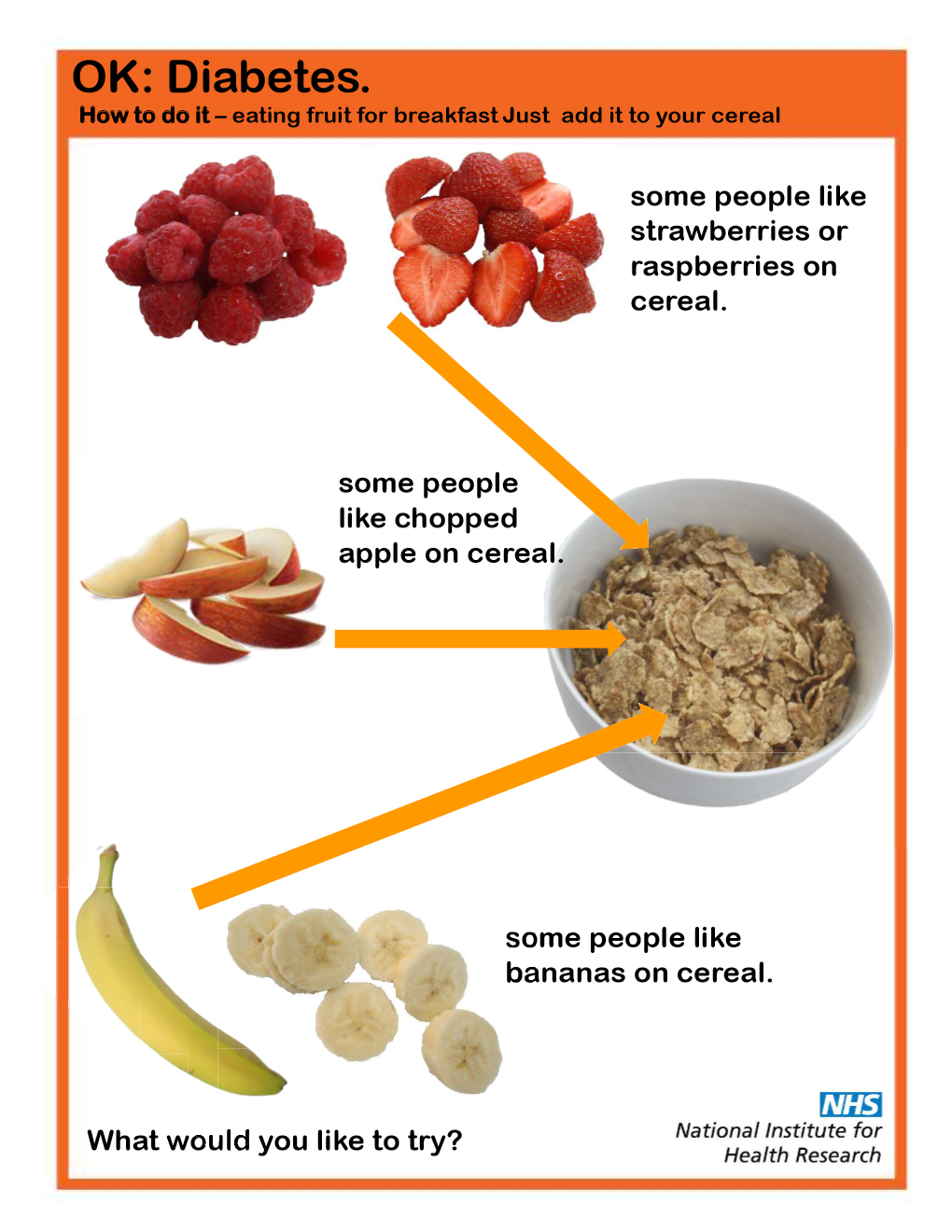


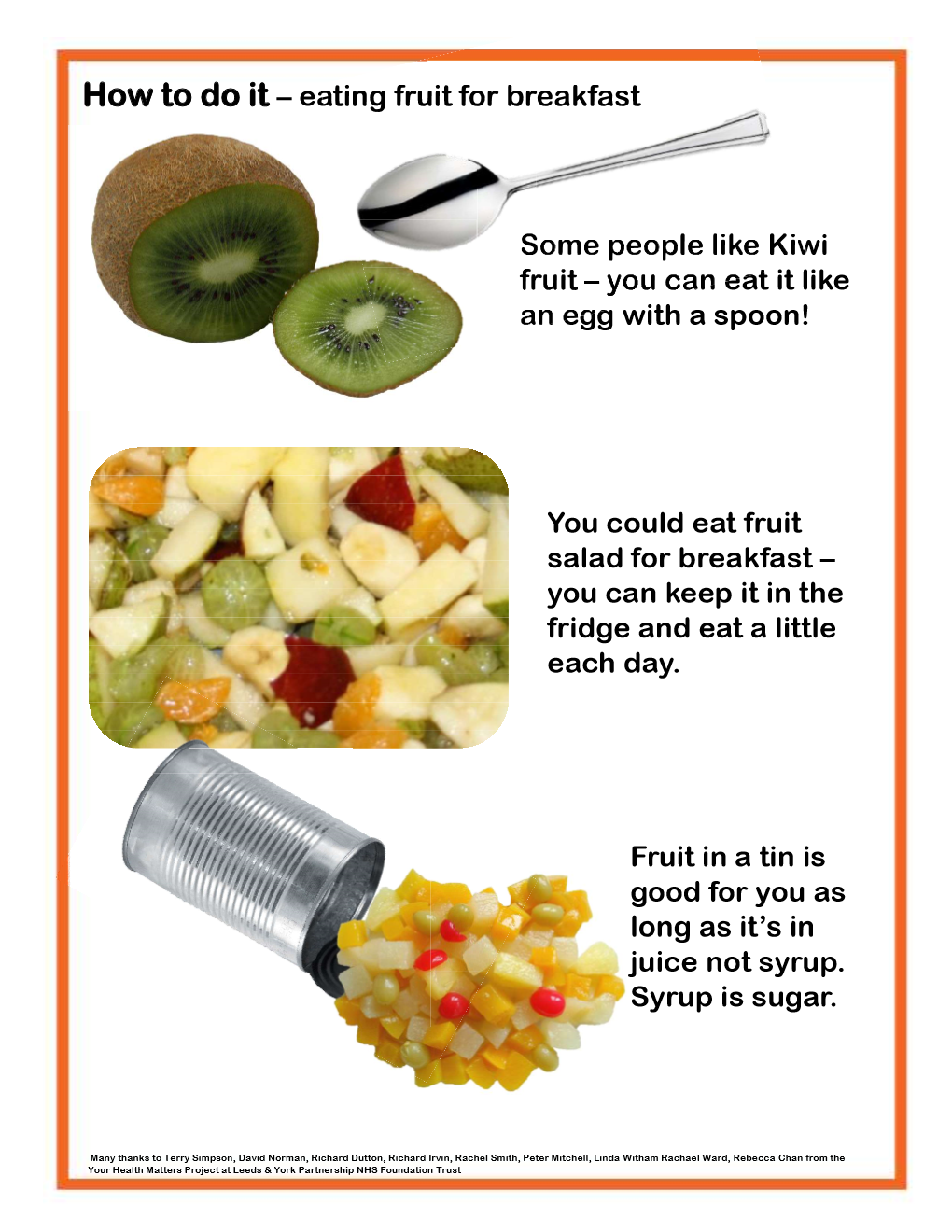


# Appendix: – how to: be more active


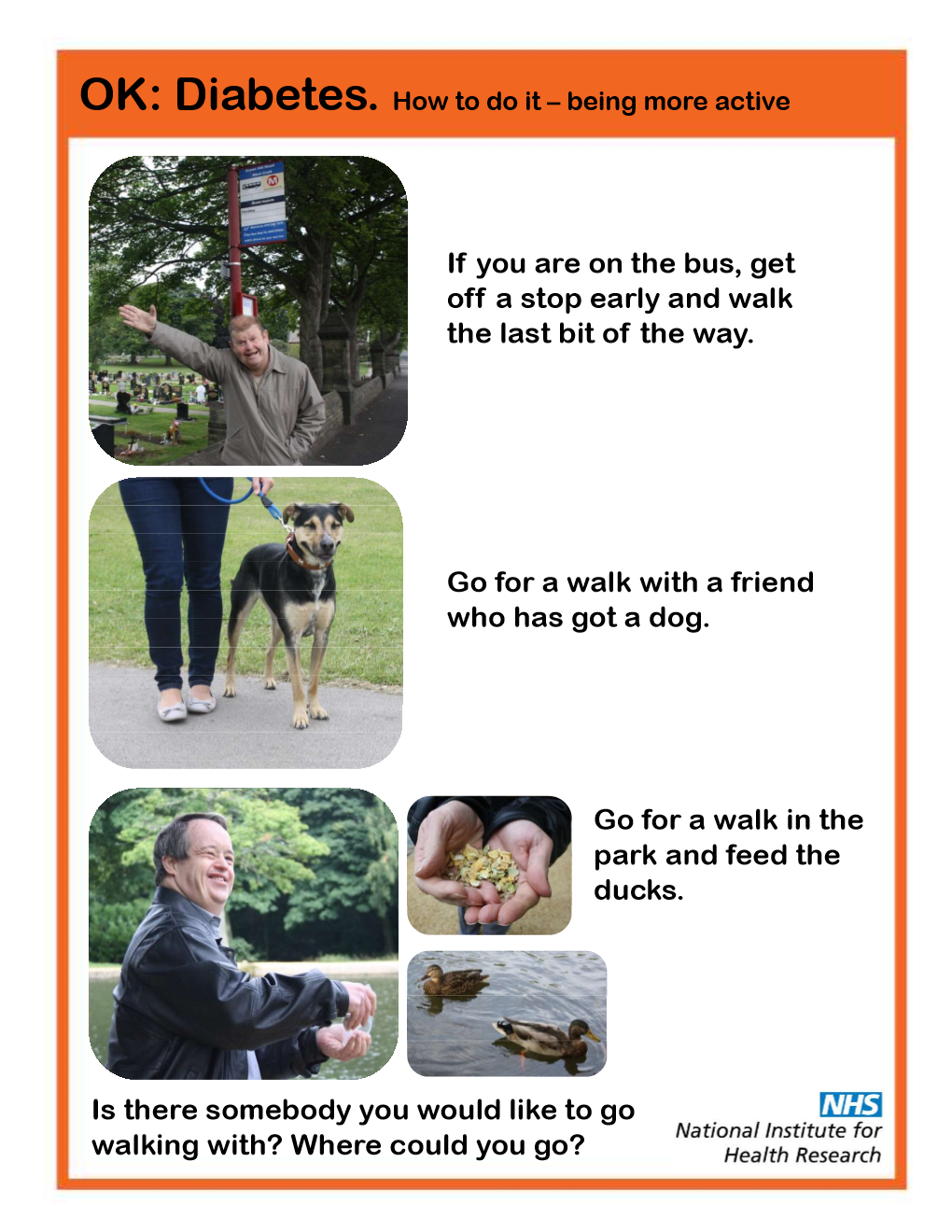


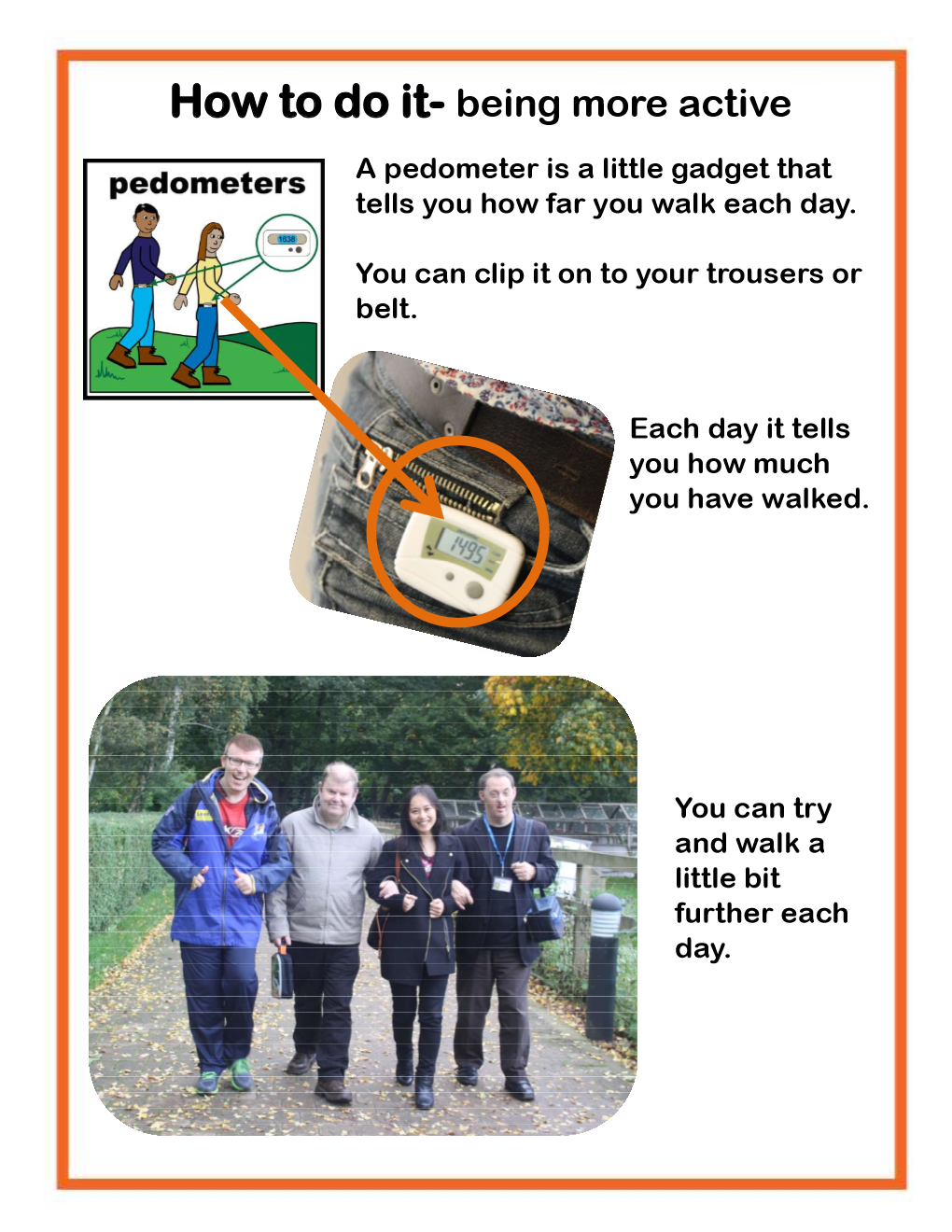


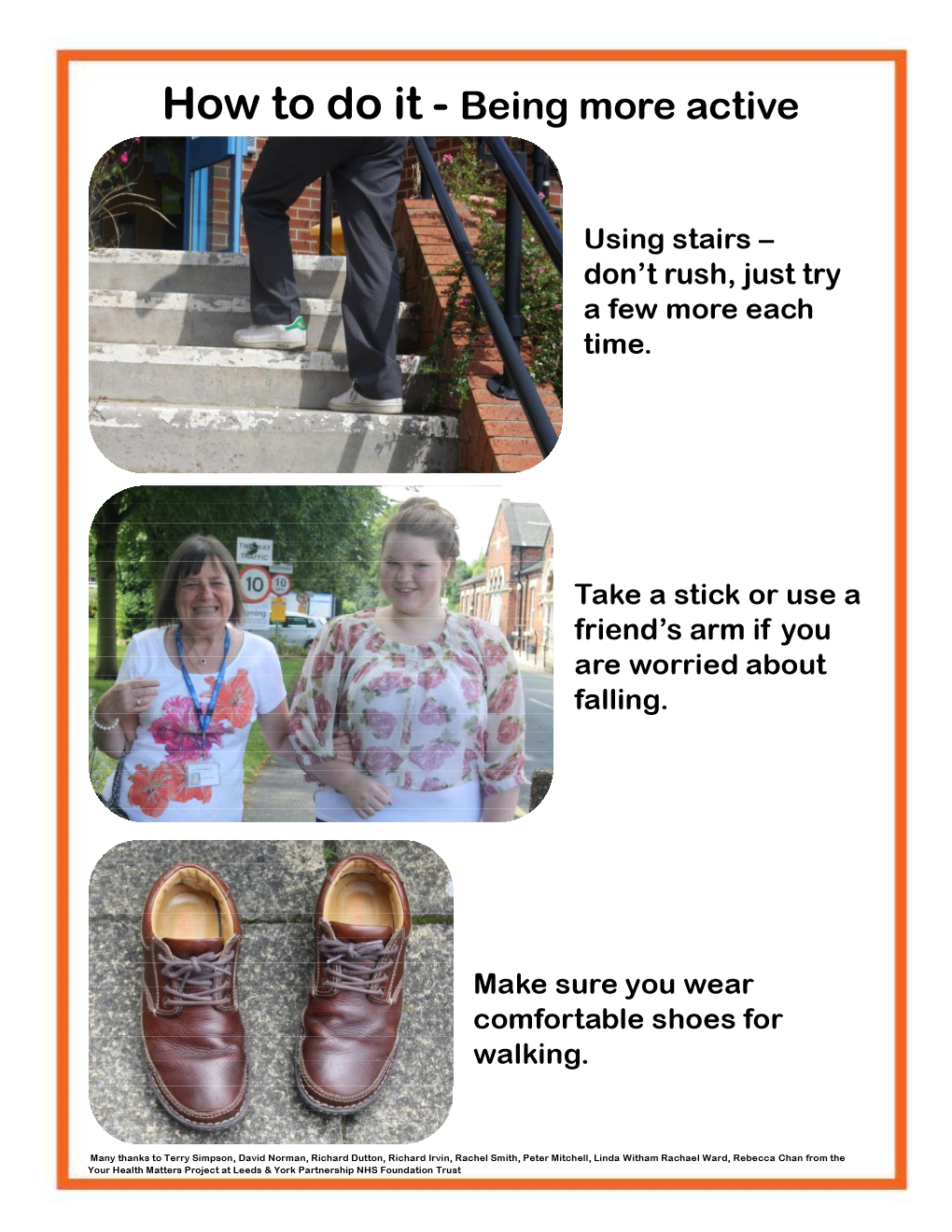


# Appendix – How to: eat more vegetables


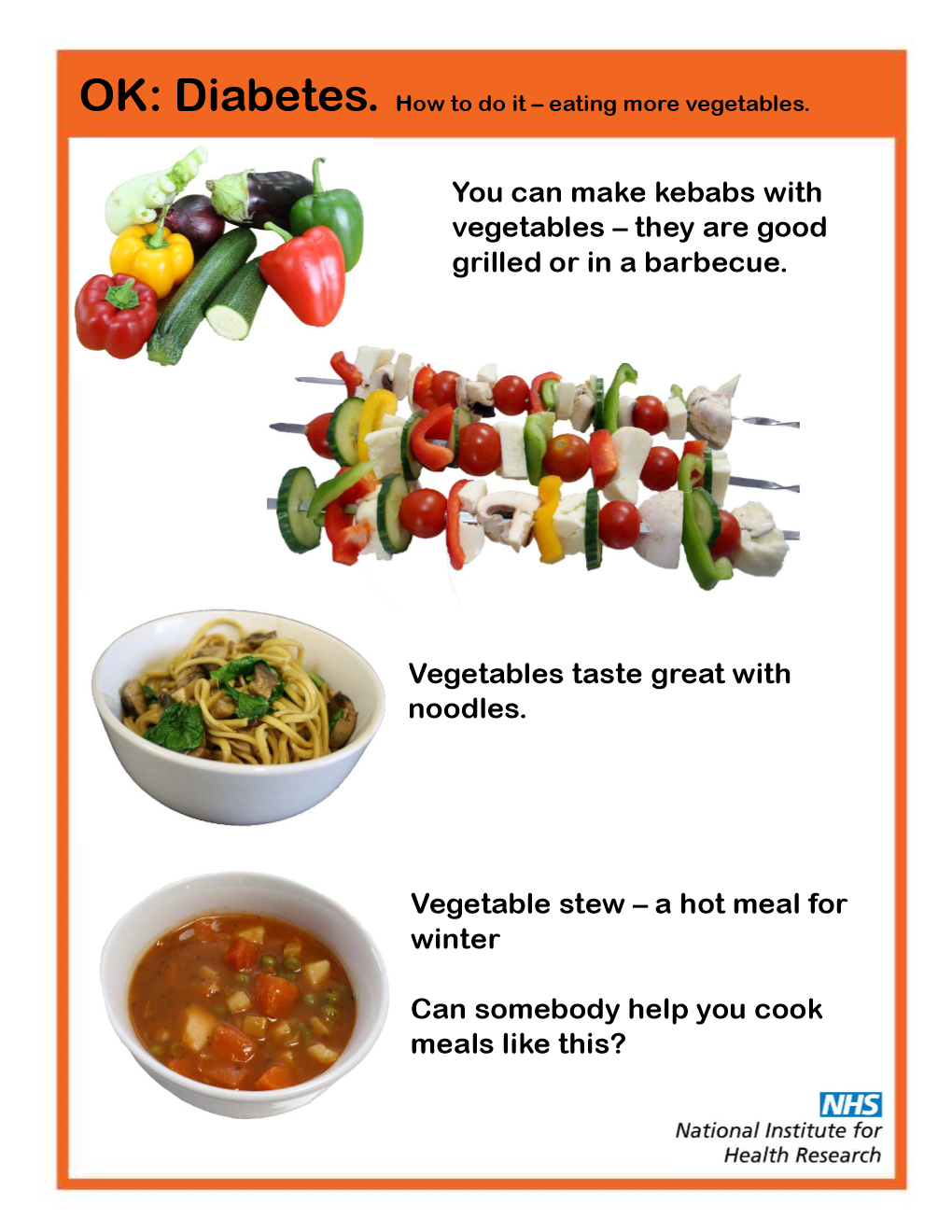


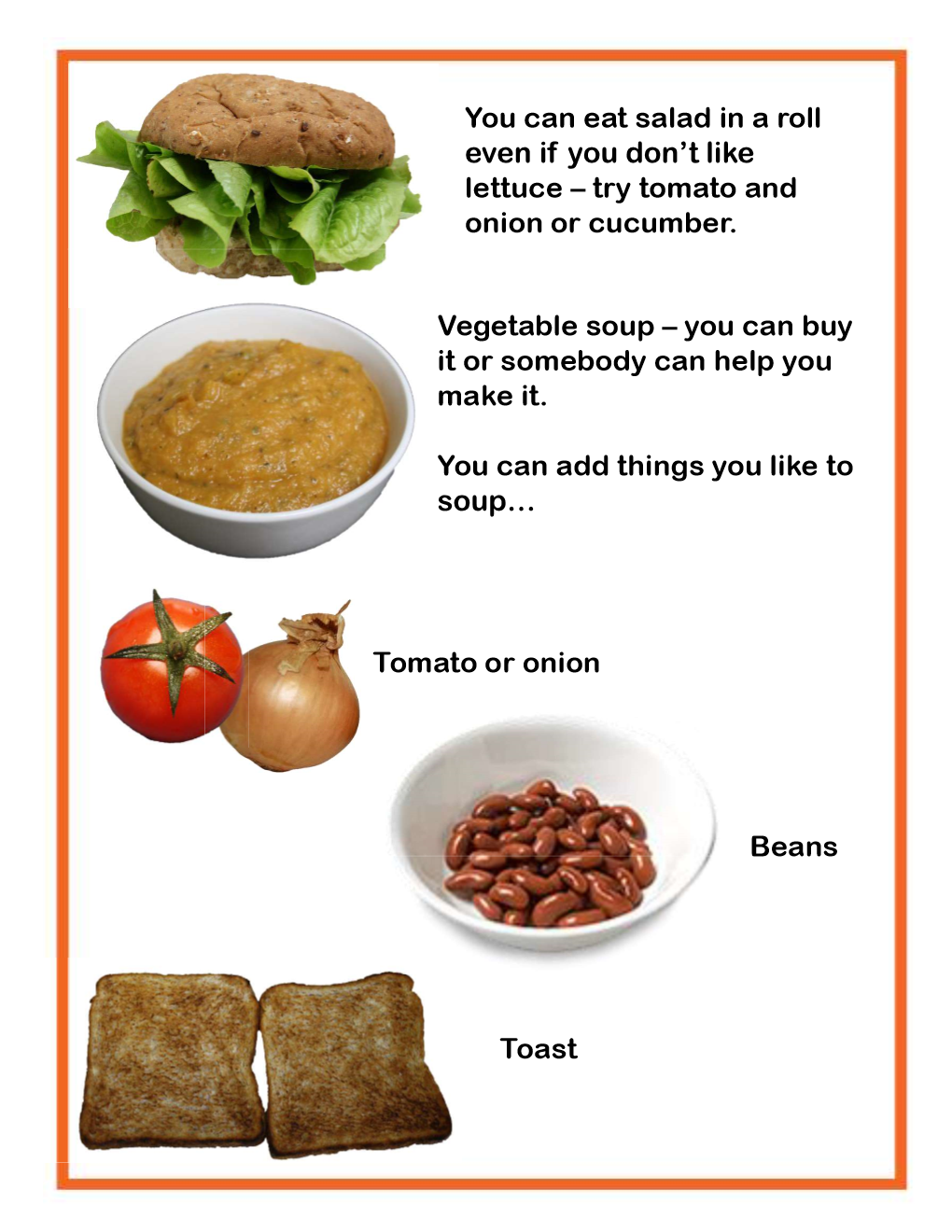


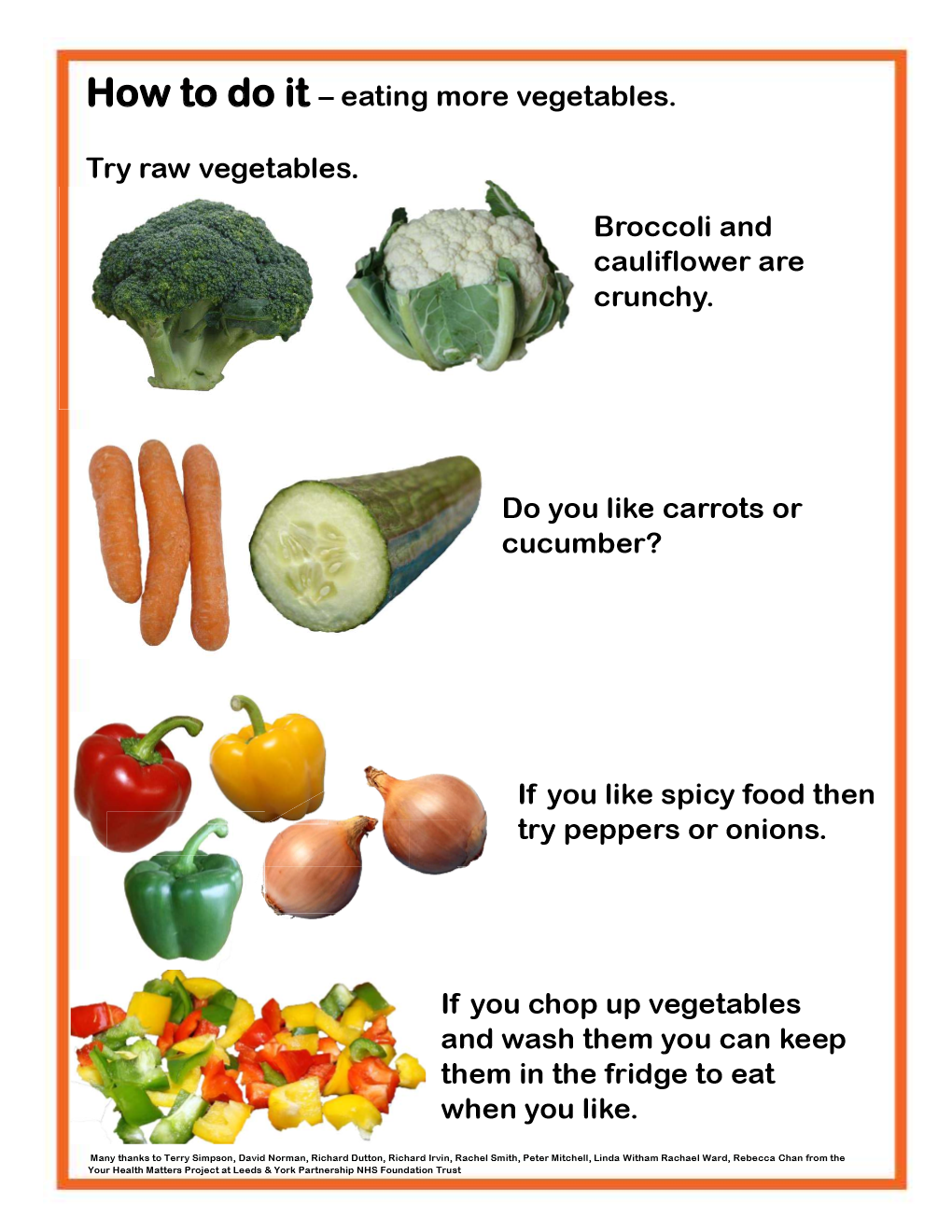

Supplement: Supplementary file 2 — How to sheet: Snack swaps. How to: eat more fruit. How to: be more active. How to: eat more vegetables. (DOCX 7684 kb) [file 40814_2018_291_MOESM2_ESM.docx]
