# Supplementary material for: Development of a supported self-management intervention for adults with type 2 diabetes and a learning disability
Source: Pilot Feasibility Stud. 2018 May 29;4:106. doi: 10.1186/s40814-018-0291-7 (PMC5975532; doi:10.1186/s40814-018-0291-7)

# Appendix Intervention materials

**OK Diabetes: supported self-management**

**A guide for nurses helping people manage their diabetes**

There are four components to the OK-Diabetes intervention. The topics in “Getting started” are likely to come up early and it may be that the best way to work is:

Getting started > setting goals > working with support > recording progress

However, as the picture suggests, the components don’t have to be delivered in a fixed order – do what suits the style of the person with diabetes.

|  |  |  |
| --- | --- | --- |
|  |  |  |
|  |  |  |

OK-Diabetes has three important characteristics:

- Uses formats that are friendly and accessible for people with learning disability
- Emphasises practical planning of actions – concentrating on simple actions for change and what might make them easier or harder to implement, rather than on education or attitudes
- Encourages a focus on the social circumstances of the person with diabetes and making use of social resources – identifying supporters and helpers who can form part of a network that supports long-term change

| **Some barriers to good diabetes care and how OK-Diabetes can help** |
| --- |
| Practical knowledge – details about what to do, what resources are available,  So... not theoretical knowledge about how you need to look after your feet and why, but what is involved in foot care and who can help. |
| Attitudes, beliefs, willingness to change, fatalism can be a (shared) problem.  So... shared choosing of feasible personal goals. |
| Tension between need for autonomy/self-control/individual responsibility and need for support, advice external structures that help.  So...establish personal goals coupled with identifying supporters who can help achieve them |
| Practical resources and logistics – money, travel difficulties, time etc.  So...always a discussion of reasons it might be difficult and who/what could make it easier |
| Physical co-morbidities may impose restrictions, symptoms (pain, fatigue); different priorities.  So...a check on mobility problems, visual impairments and other difficulties that mean goals need to be modified |
| Lack of social and family support e.g. instrumental (cues to action, encouragement, practical assistance) or emotional (marital and other distress makes it harder)  So... a strong emphasis on identifying supporters and other helpers in the social network |
| Emotional factors – diabetes-related helplessness, frustration  So...questions about emotional state as well as physical problems |
| Lack of support from healthcare system  So...an understanding that self-management includes knowing how to use the healthcare system |
| An environment that supports healthy routines and habits  So...identifying day-to-day routines as well as diabetes regime |

**The aims of “getting started” are:**

- Starting to get to know the person with diabetes – understanding their general lifestyle and social network
- Finding out about the person‘s life outside diabetes, and what they like to do
- Making an assessment of the diabetes care and health state of the person with diabetes
- Discuss diabetes and possible ideas for change
- Identify and name key supporters and other helpers

**First steps**

- - You may want to show a badge or picture of yourself
  - Remind the person about the project and previous contact in Phase 1 (you could ask if they still have Phase 1 leaflet),
  - Check that the consent form applies and that they still give consent.

| **Action** | **Materials** | **Record actions (examples)** | **Confirmed**[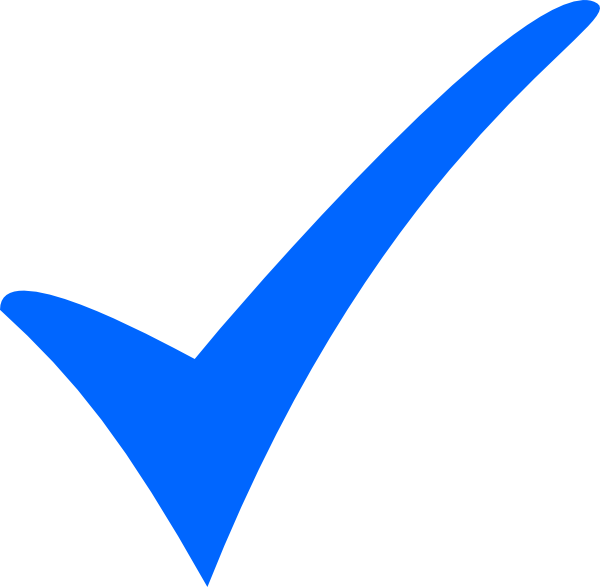](http://www.clipartbest.com/clipart-RiA6jzMxT) |
| --- | --- | --- | --- |
| Introduction; explanation of project | Personal ID  *<Phase 2 Information sheet>* | *Shown to participant*  *Copy left with participant* |  |
| Check consent | *<Consent form>* | *Checked and confirmed for CTRU* |  |

Notes:

**Getting to know you**

Suggested opening – “I want to know a bit about you and what you do and who you see, to help us think about how to help you look after your diabetes”

“Can you tell me a bit about how you spend your week? Are there places you go to every week? Do you work, or go to a Day Centre?

What sort of things do you like doing? Are there thing you don’t like doing or that worry you?

What about friends and family? Who do you see?

| **Actions** | **Materials** | **Recorded actions** | **Confirmed** [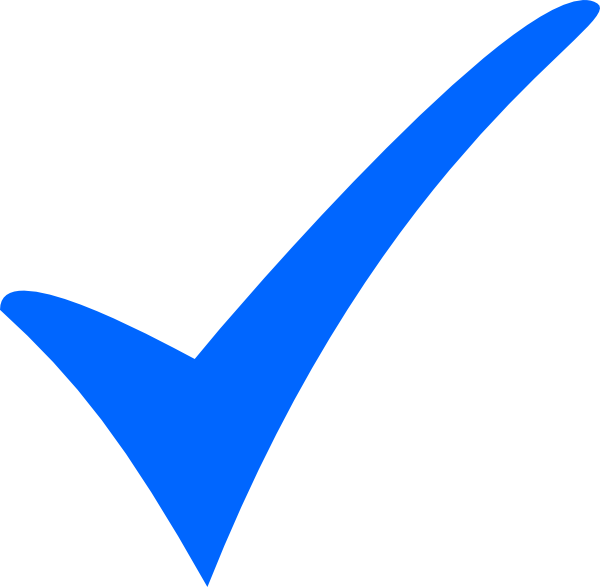](http://www.clipartbest.com/clipart-RiA6jzMxT) |
| --- | --- | --- | --- |
| Understanding | *<My week>* – timetable  *<My life>* - chart  <*My friends + family> - sociogram* | *Weekly timetable and My Life chart – completed and copies in folder* |  |

Notes:

**Your diabetes**

Review main aspects of diabetes; start with the Looking After my Diabetes chart or use the Phase 1 interview to cover the main areas – diet; activity; medication, foot care and so on.

| **Action** | **Materials** | **Recorded actions** | **Confirmed [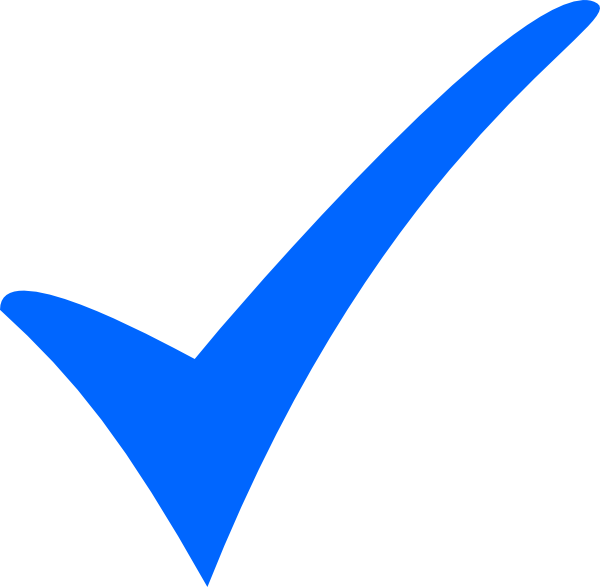](http://www.clipartbest.com/clipart-RiA6jzMxT)** |
| --- | --- | --- | --- |
| Discuss diabetes with person with diabetes | *<Phase 1 assessment>*  *<Looking after my diabetes>* | *Interview completed and in folder. “Looking after my diabetes”* |  |

Notes:

**Starting to identify supporter and other helpers**

At this stage, it is worth checking that you have an idea of who helps with various aspects of diabetes self-management, and identifying any named helpers who might be able to

“We’ve mentioned a few people you know (family, friends, others). Can I just check that I understand who helps even in small ways with your diabetes – what you eat, what you do generally and not just your diabetes care”

NOTE: people use terms like supporter, helper, carer differently. In OK-Diabetes each person has only ONE supporter – the person who provides most support in day-to-day life and looking after diabetes. The supporter will have been identified in Phase 1 and at consent for Phase 2. Check who it is. We call all other involved people “helpers”

| **Action** | **Materials** | **Actions recorded** | **Confirmed[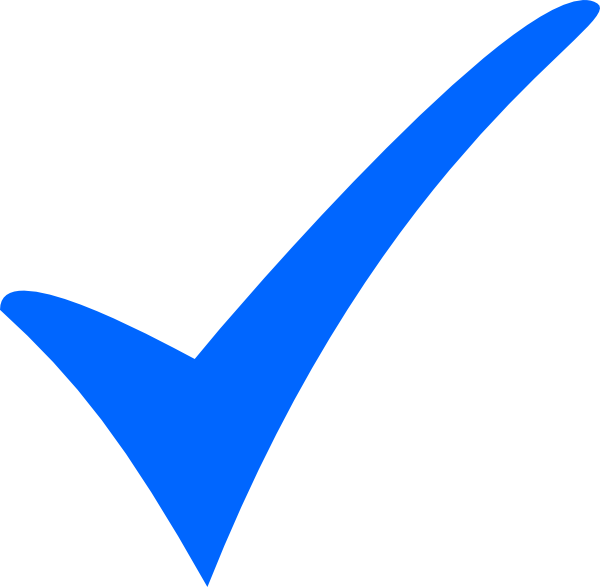](http://www.clipartbest.com/clipart-RiA6jzMxT)** |
| --- | --- | --- | --- |
| Clarify “key supporter(s)” and “other helpers” | *<My supporters and helpers>* - Checklist | *Named supporters identified and in folder* |  |

Notes

**Thinking about goals**

Finally, if there is time, start a discussion about areas of self-management the person with diabetes might like to change. At this stage it is a very general discussion about possibilities. Nothing needs to be planned definitely or agreed – just get an idea

“What might you like to try that’s different? Are you happy with what you eat? How much exercise you take?” (Some gentle prompts are in order but be careful not to instruct). Who might help? What would make it hard to change?

NOTE: They will have had this discussion during Phase 1 – it is worth checking the last part of Form 10 to see what they said then.

| **Action** | **Materials** | **Actions recorded** | **Confirmed[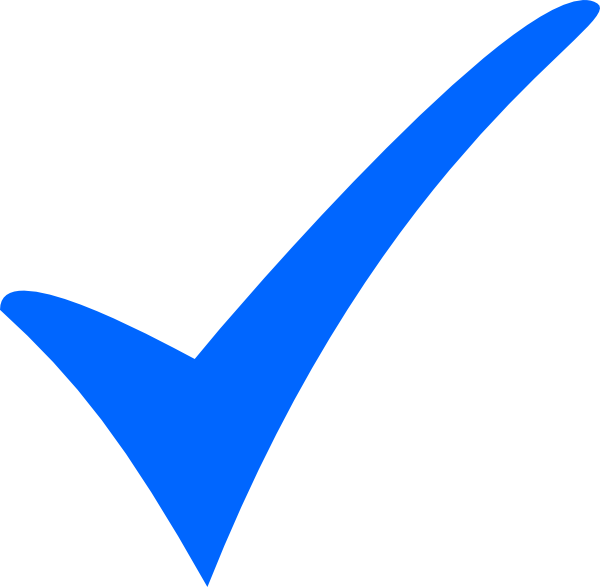](http://www.clipartbest.com/clipart-RiA6jzMxT)** |
| --- | --- | --- | --- |
| Establish initial possibilities for change, think about what might support or impede change | *<What where when>* - Chart | *Possible action points identified for goal setting* |  |

Notes:

Getting started: further notes

After “Getting started”: the person with diabetes will:

- know nurse,
- know what OK-Diabetes is for and will have consented
- described daily life
- discussed main aspects of diabetes
- starting thinking about ideas for change
- have a “My OK-Diabetes Space”
- know what the next step is – another appointment for example (use A Note For...).

NOTE: set up “My OK-Diabetes space” now,

- Add your contact details
- Use “a note for” to add next appointment

**The aims of “Setting goals” are:**

- Building on initial ideas for change, identify one or two definite goals for change
- Choose a goal and define it clearly
- Provide relevant materials
- Identify people and things that will make the behaviour easier and more difficult to change
- Make a detailed action plan for the first goal

**NOTE**: You may want to run this straight into <**Recording Progress**> depending upon how the discussion goes

**First off – a recap:**

Check main details from “Getting started” and confirm ideas about change. Make sure this is really something the person wants to do and not something they are just saying to agree with what is expected -

“When you talked to a researcher before you thought you might like to ....Is that still true? Ask questions about reasons for change, what they can imagine being better and so on.”

Agree the change to start working on and label <I am going to> box accordingly.

| **Action** | **Materials** | **Actions recorded** | **Confirmed [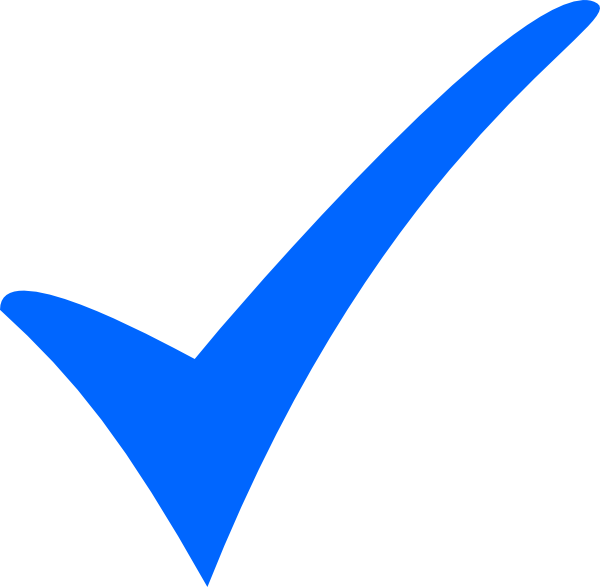](http://www.clipartbest.com/clipart-RiA6jzMxT)** |
| --- | --- | --- | --- |
| Review candidates for change | <*what where when> chart* | *Chart reviewed and agreed or changed* |  |
| Identify preferred goal for change | *<I am going to>* - box | *First goal agreed. < I am going to > box started and copy for participant* |  |

Notes:

**Agreeing more details about a goal**

- Try to specify in a bit more detail what the desired action will be e.g. walking for ½ hour 3 times a week; eating an extra portion of fruit a day; not eating biscuits when watching TV in the evening. This is better than general plans like “eat fewer sweet things” or “get fit”
- Provide <How to do it> sheets or other materials that might help with ideas, discuss and come to an agreement:

Ask about

- Eating more fruit
- Eating more vegetables
- Snack swap
- Fizzy drinks
- Physical activity
- Health checks – feet, teeth, eyes

NOTE!! It is extremely easy to get “pseudo-agreement” to a change decided by somebody else. Think of this step as a starter to exploring what might really be wanted by the person with diabetes.

| **Action** | **Materials** | **Actions recorded** | **Confirmed [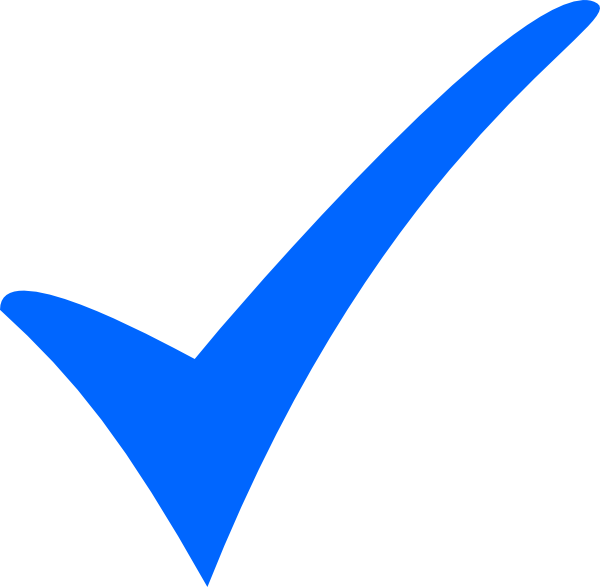](http://www.clipartbest.com/clipart-RiA6jzMxT)** |
| --- | --- | --- | --- |
| Define and specify action towards goal | *<How to do it> -* Educational materials on diet, physical activity, foot checks etc | *Goal set and recorded in <I am going to box>*  *Relevant sheets given* |  |

Notes:

**Turning a goal into an action plan**

- Identify a detailed plan for how to do it – what to do when, who will help, what else might be needed to make it work. Use
- Rehearse how it might go = think about problems as well. Use the chart to think hard with the person about what might make it difficult. Use prompts, give permission for people to say they have tried and failed before, or it seems too difficult.
- Here is another good opportunity to look at the supporters/helpers list

| **Action** | **Materials** | **Actions recorded** | **Confirmed [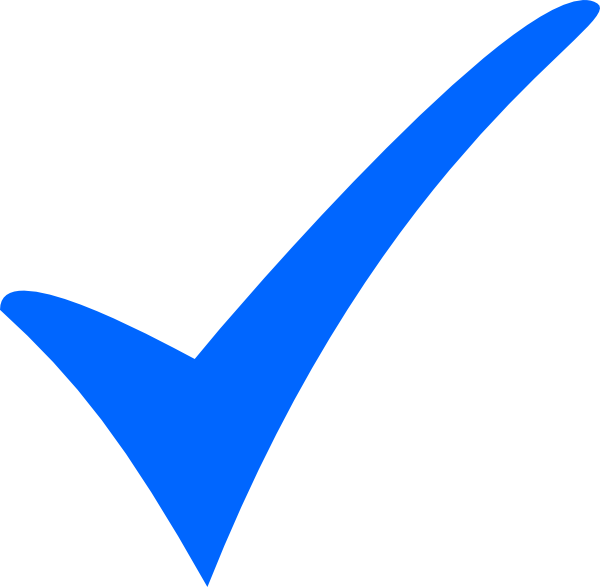](http://www.clipartbest.com/clipart-RiA6jzMxT)** |
| --- | --- | --- | --- |
| Identify people and resources needed to support change | *<I am going to>*- chart | *Revisited and edited who/what/where with specific goal in mind* |  |

Notes:

**Recording an action plan**

This involves recording a step-by-step plan of action for the chosen goal: I will do xx (when, where) I will ask yy to do zz to help and so on. This action plan should be recorded in the <I am going to> plan with copies for the person with diabetes, the nurse and any named supporters

| **Action** | **Materials** | **Actions recorded** | **Confirmed [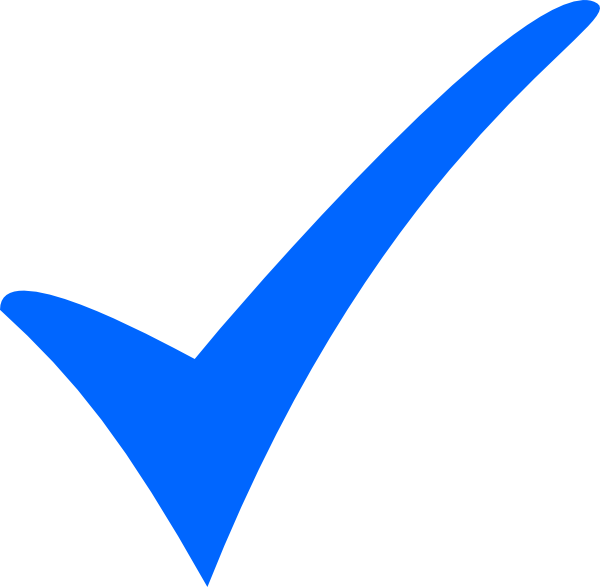](http://www.clipartbest.com/clipart-RiA6jzMxT)** |
| --- | --- | --- | --- |
| Record a specific action with detailed steps | *<I am going to>*- plan | *<I am going to> plan completed and copy left with participant* |  |

Notes:

Setting Goals: further notes

After “Setting goals”: the person with diabetes will:

- Have decided on something to change,
- Have been given any materials that may help
- Have discussed the main help needed
- Agreed and written down an action plan – may be Box, Chart or Plan initially depending how far you get
- know what the next step is – another appointment for example.

Add to OK Diabetes space –

“I am Going to box” or “I am Going to plan”

**The aims of “Working with support” are:**

- To identify a key named supporter who will help with action plans
- To identify other helpers who will help with action plans

**Starting with the person with diabetes:**

- Identify all the people they see at least every week, or live with, who are important as friends or family.
- Review the <Who What Where> chart, the sociogram and the <supporters and helpers checklist> to make sure that everybody has been identified
- Check who could help

| **Action** | **Materials** | **Actions recorded** | **Confirmed [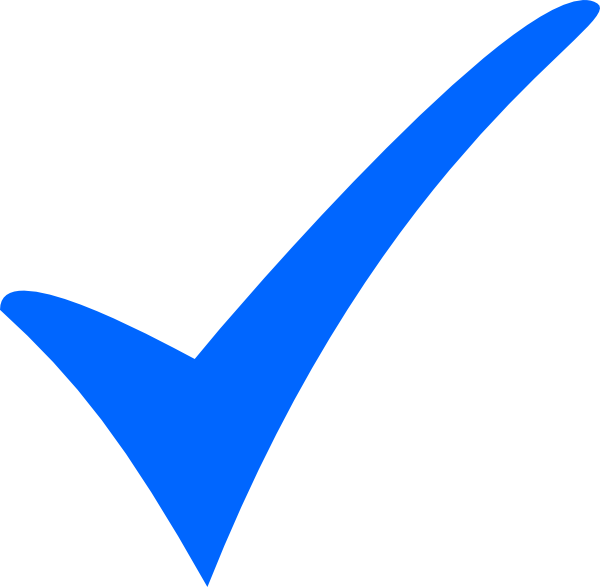](http://www.clipartbest.com/clipart-RiA6jzMxT)** |
| --- | --- | --- | --- |
| Identify the household, family and friends of the person with diabetes | *<Sociogram>* | *Sociogram completed and filed* |  |
| Identify potential helpers in the diabetes action plan | *<Who what where>*- chart | *<Who what where>*- chart reviewed |  |
| Further check on the supporters and helpers checklist |  | *<supporters and helpers checklist>* reviewed |  |

Notes:

**Contacting the supporter and other helpers:**

You should try always to find at least ONE, ideally 2-3 people who will help

***For each person you need to ask***

“Do you think they would like to help you?”

“How shall we involve them?”

- speak to them,
- write,
- leave note with the person with diabetes
- arrange to meet together next time (of course a supporter may already be present)

NOTE – on the supporter and helpers checklist who you will contact and how, and what they will be asked to do (you could fill out a dummy flashcard)

**Putting people in touch with other helpers**

You should consider local resources – how can you put people in contact with a statutory or 3^rd^ sector helper –

Make a note HERE of any such suggestions

| Other organization/help suggested | Action taken |
| --- | --- |
|  |  |

Notes:

Consider – referral for assessment for a personal health budget

**Meeting with named helpers (complete for each helper or supporter contacted)**

- Check that they agree with their place in the social network
- Check that they are willing to help
- Explain OK-Diabetes and provide information sheet and supporter’s pack
- Give copy of I am Going To chart and explain
- Give copy of supporter flashcard and discuss their role

| **Action** | **Materials** | **Actions recorded** | **Confirmed [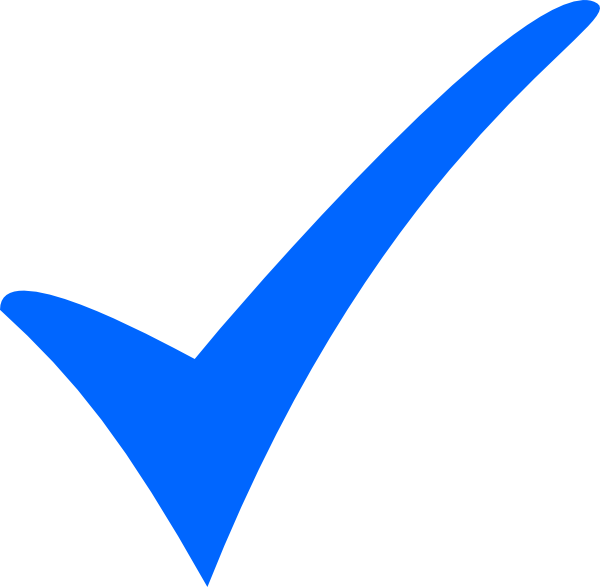](http://www.clipartbest.com/clipart-RiA6jzMxT)** |
| --- | --- | --- | --- |
| Ensure named helpers are accurately identified and understand OK-Diabetes | *<Sociogram> <Who What Where> <Information Sheet> <Supporters Pack>* |  |  |
| Ensure named supporters understand the action plan | *<I am going to>*- plan  *<Flashcard>* |  |  |

Notes:

**Helpers from statutory or 3^rd^ sector organizations**

**In addition to the above…**

- **For some supporters** eg employees of care providers
- Discuss training needs
- Ask about recognition eg certificate

| **Action** | **Materials** | **Actions recorded** |
| --- | --- | --- |
| Ensure named helpers are appropriately recognized and supported | *<Supporters Pack>*  *Bespoke letter of thanks or certificate* |  |

Notes:

After “Mapping support”: the person with diabetes will:

- Have identified all the key people in their social network,
- Have named their supporters for diabetes

After “Working with support”: named helpers will:

- have been seen and given any materials about OK-Diabetes
- will have discussed their role in any help needed
- will have discussed their own needs for support and recognition

All parties will:

- know what the next step is – another appointment for example.

**The aims of Recording Progress are:**

- Establish the OK Diabetes Space as somewhere the person with diabetes keeps a clearly visible reminder of their “I am going to” plans
- Establish the OK Diabetes Space as somewhere the person with diabetes records whether they have achieved their goals each day
- Establish a system for collecting completed records of actions, to assist in monitoring adherence.

**What happens**

- Ensure the “I am Going to” plan is available, understood by the person with diabetes, and posted in the OK-Diabetes Space
- Use “How to Do it” sheets, helper flashcards and other resources to rehearse how it will work
- Provide “Calendar Sheets” and explain how to use them to record actions
- Provide box for collection of Calendar Sheets
- AGREE who will support person with diabetes in remembering to complete this task every day

| **Action** | **Materials** | **Actions needed** |
| --- | --- | --- |
| Agree how actions to meet goal will be recorded | I am Going to..plan + helper flashcards for OK Diabetes Space |  |
| Agree daily record | Calendar Sheets  Modify chart |  |
| Provide materials for recording progress | Box for calendar sheets  Pens |  |

Notes:

IMPORTANT: you must make a follow up visit at 4 weeks after this system is established = a top-up/revision and to collect the Calendar Sheets

After “Recording Progress”: the person with diabetes will:

- Know how to record progress against their action plan
- Know who will help them record progress
- Know how they will measure success ,
- know what the next step is – another appointment for example.

Example of OK Diabetes ‘Space’ in use as part of the intervention


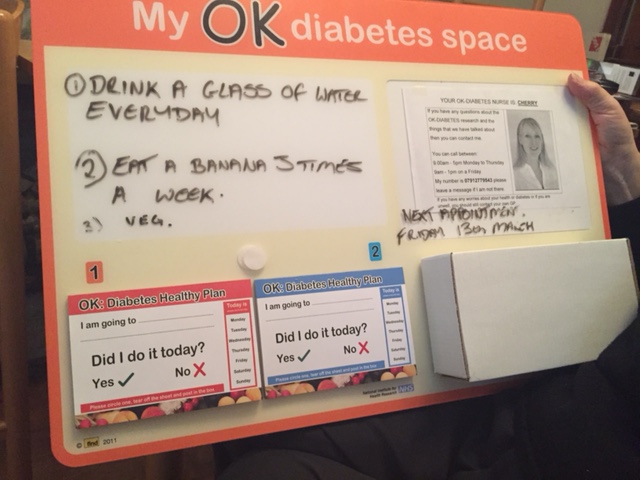

Supplement: Supplementary file 3 — Intervention materials. (DOCX 209 kb) [file 40814_2018_291_MOESM3_ESM.docx]
